# Brain functional connectivity analysis in patients with relapsing-remitting multiple sclerosis: A graph theory approach of EEG resting state

**DOI:** 10.3389/fnins.2022.801774

**Published:** 2022-09-09

**Authors:** Sepehr Shirani, Maryam Mohebbi

**Affiliations:** ^1^Department of Biomedical Engineering, Faculty of Electrical Engineering, K. N. Toosi University of Technology, Tehran, Iran; ^2^Department of Computer Science, Nottingham Trent University, Nottingham, United Kingdom

**Keywords:** EEG, functional connectivity, graph theory, independent components, multiple sclerosis

## Abstract

Multiple sclerosis (MS) is an autoimmune disease related to the central nervous system (CNS). This study aims to investigate the effects of MS on the brain's functional connectivity network using the electroencephalogram (EEG) resting-state signals and graph theory approach. Resting-state eyes-closed EEG signals were recorded from 20 patients with relapsing-remitting MS (RRMS) and 18 healthy cases. In this study, the prime objective is to calculate the connectivity between EEG channels to assess the differences in brain functional network global features. The results demonstrated lower cortical activity in the alpha frequency bands and higher activity for the gamma frequency bands in patients with RRMS compared to the healthy group. In this study, graph metric calculations revealed a significant difference in the diameter of the functional brain network based on the directed transfer function (DTF) measure between the two groups, indicating a higher diameter in RRMS cases for the alpha frequency band. A higher diameter for the functional brain network in MS cases can result from anatomical damage. In addition, considerable differences between the networks' global efficiency and transitivity based on the imaginary part of the coherence (iCoh) measure were observed, indicating higher global efficiency and transitivity in the delta, theta, and beta frequency bands for RRMS cases, which can be related to the compensatory functional reaction from the brain. This study indicated that in RRMS cases, some of the global characteristics of the brain's functional network, such as diameter and global efficiency, change and can be illustrated even in the resting-state condition when the brain is not under cognitive load.

## Introduction

Multiple sclerosis (MS) is an autoimmune disease with disseminated cortical and white-matter lesions and devastating consequences for the patients (Dendrou et al., 2015). In the nervous system, axonal fibers provide the anatomical basis for signal transfer and communication between different regions. The damaged myelin causes communication problems between different regions of the CNS. Further processes of remyelination and structural and functional rearrangement are hallmarks of the disease (Filippi and Agosta, 2009). Focal demyelination is spread over the entire brain, primarily involving white matter, with predilection sites in the periventricular regions. In contrast, gray matter lesions are increasingly considered significant trademarks of the disease and play an essential role in the long-term functional outcome (Seewann et al., 2011; Droby et al., 2015, 2016). Due to the damaged axonal fibers and communication problems between various regions, patients with MS face severe daily life challenges. Generally, patients with MS show sensory impairments including pain and unpleasant sensations; an imbalance that can arise from cerebellar dysfunction, cognitive impairments, and slowed information processing; depression that may relate to injury of frontotemporal networks; fatigue, bladder, and bowel dysfunction; heat sensitivity; headache; and pseudo relapse. Furthermore, brain stem syndromes such as double vision and facial weakness are common in these patients. Relapsing-remitting MS (RRMS) is the most common form of MS in which relapses are discrete periods of neurologic dysfunction that commonly evolve over hours to days and then persist for days to weeks before remitting. In between relapses, patients are generally stable but still often experience fatigue and heat sensitivity (Gelfand, 2014). Previous studies have used multimodal evoked potentials as a robust monitoring tool to compare the ability of different evoked responses to investigate and predict the disease course (Schlaeger et al., 2016).

In addition to task-directed studies, another approach to investigate brain activity is to gather data during the resting state when the subjects' nervous system is not responding to a specific stimulation (Biswal et al., 1995). In previous years, some studies have focused on monitoring brain activity in patients with MS during the resting state to investigate the abnormalities using fMRI to reach a deeper understanding of the impacts of MS on the brain's function (Liu et al., 2011; Tahedl et al., 2018; Saccà et al., 2019). However, although neuroimaging methods such as MRI and fMRI have a high spatial resolution due to their data acquisition method, they are usually expensive for continuous recordings. Continuous recordings help researchers to monitor disease progression or examine the effects of different treatments on patients for an extended period. Furthermore, using fMRI in studying the brain's function can be time-consuming, which can cause various problems for patients with MS due to their physical condition, making the data acquisition process arduous. Moreover, another problem with fMRI-based methods is that they have a low temporal resolution that can lead to information loss, which is not ideal for monitoring the activity of a dynamic system like the brain.

Electroencephalography can be a decent method to monitor brain function as its recording cost is less than other neuroimaging methods. In previous years, researchers have investigated EEG resting-state to monitor the brain's cortical activity and compare the brain activity differences between healthy people and MS phenotypes in different frequency bands (Babiloni et al., 2016). The results of these studies show differences in the activity of various frequency bands between MS phenotypes and healthy cases in various cortical regions.

Mapping the active functional properties of brain networks is a crucial goal of basic and clinical neuroscience and medicine. Brain network theory has been repeatedly used to understand brain functions. This theory explains that the brain's complex functions result from the communication between different brain regions (Lang et al., 2012; Sporns, 2016). One of the powerful tools for quantifying and analyzing the characteristics of connectivity networks is graph theory. Graph theory has become one of the most useful methods for modeling brain networks as interconnected systems in recent years (Bassett and Sporns, 2017). The graph theory approach has been proven worthy in various brain network research for subjects with both normal and pathological conditions such as major depressive disorder and Alzheimer's disease (Brier et al., 2014; Hasanzadeh et al., 2020). Network science contributes powerful access to primary organizational principles of the human brain, and it has been applied with graph theory to distinguish brain structural and functional connectivity patterns in MS (Fleischer et al., 2019). Previous studies have used the graph theory approach to investigate structural connectivity in MS and introduce methods to classify patients with MS in several clinical profiles (Kocevar et al., 2016). fMRI and MEG-based neuroimaging studies have used the graph theory approach for studying functional connectivity in MS to assess impaired functional integration and characterize topological patterns of connectivity changes (Schoonheim et al., 2013; Dogonowski et al., 2014; Rocca et al., 2016).

According to previous EEG studies that investigated brain activity during resting state in MS cases, we hypothesize that EEG resting-state can show significant differences in brain functional network characteristics between MS and healthy cases. To validate this hypothesis, we constructed the brain network by calculating connectivity measures between EEG channels after preprocessing. In this study, EEG-based networks have been constructed to compare the brain network between RRMS and healthy cases. The directed transfer function (DTF) and imaginary part of coherence (iCoh) were chosen to investigate the information flow and their activity synchronization. The rest of this study has been organized into four sections: first, in the “Participants and method” section, we describe the subjects who participated in this research, then the EEG data recording and preprocessing procedure are explained. Next, we describe the method used for constructing brain networks, and then graph theory features extracted from the networks are mentioned. Next, in the “Result” section, alterations in the networks of patients with MS are represented, and the two groups' network characteristics are compared. Then, the obtained results are discussed in the “Discussion” section. Finally, this study is concluded in the “Conclusion” section.

## Participants and method

### Participants

For this study, 20 patients with MS and 18 healthy cases were selected. All patients were diagnosed with relapsing-remitting MS and had a similar level of disability. RRMS is the most common initial form of MS. It is described as MS, in which patients have relapses of the disease and periods of stability in between relapses. A neurologist assessed all patients using the expanded disability status scale (EDSS) (Kurtzke, 1983), which ranges from 0 to 10 in 0.5-point increments and measures the overall disability level. The EDSS ranged from 3 to 4 for all patients (12 with EDSS = 3, 6 with EDSS = 3.5, and 2 with EDSS = 4). The cases showed mild to moderate impairment in the form of muscle weakness, numbness, and a problem with balance, with more severity for cases with higher EDSS. Of 20 patients, 2 cases were reported to have occasional remembering problems, and 3 were reported with occasional sight problems. All the patients were in the remitting period of their disease during data collection. The Wilcoxon rank-sum test compared the gender and age of the participants in the two groups, and the two groups were closely matched. All participants provided written informed consent, and the Iran Medical University ethics committee approved the study, and we made sure that the participation process was comfortable for them. The approval number for the study is “IR.IUMS.REC.1395.9406414.” Tables 1, 2 show the information of participants. The data that support the results of this research was recorded at the National Brain Mapping Laboratory (NBML).

**Table 1:** The gender and the average age of participants.

**Group**	**Gender**	**Average age**
20 RRMS patients	13 females + 7 males	34.25 ± 10.2 years
18 Normal subjects	11 females + 7 males	34.5 ± 9.4 years

**Table 2:** The information related to the condition of RRMS cases.

**Case number**	**Gender**	**EDSS**	**Impairments (In order of severity)**
Case-1	Female	3	Muscle weakness + numbness
Case-2	Female	3	Numbness + muscle weakness + having problem remembering
Case-3	Male	3	Muscle weakness + occasional sight problem
Case-4	Male	3	Muscle weakness + numbness
Case-5	Female	3	Muscle weakness + problem with balance
Case-6	Female	3	Muscle weakness + numbness +occasional sight problem
Case-7	Female	3	Muscle weakness + problem with balance
Case-8	Female	3	Muscle weakness + problem with balance
Case-9	Male	3	Numbness + problem with balance
Case-10	Female	3	Muscle weakness + problem with balance
Case-11	Female	3	Muscle weakness + problem with balance + numbness
Case-12	Female	3	Muscle weakness + numbness + mild problem with balance
Case-13	Male	3.5	Moderate muscle weakness + problem with balance + mild numbness
Case-14	Female	3.5	Moderate muscle weakness + problem with balance
Case-15	Male	3.5	Moderate numbness + muscle weakness + occasional sight problem
Case-16	Female	3.5	Moderate problem with balance + muscle weakness
Case-17	Male	3.5	Moderate problem with balance+ muscle weakness + having problem remembering
Case-18	Male	3.5	Moderate muscle weakness + problem with balance
Case-19	Female	4	Moderate muscle weakness and numbness + problem with balance (able to walk by herself, self-sufficient in daily tasks)
Case-20	Female	4	Moderate muscle weakness and numbness + problem with balance (able to walk by herself, self-sufficient in daily tasks)

### EEG recordings

**Figure 1:**
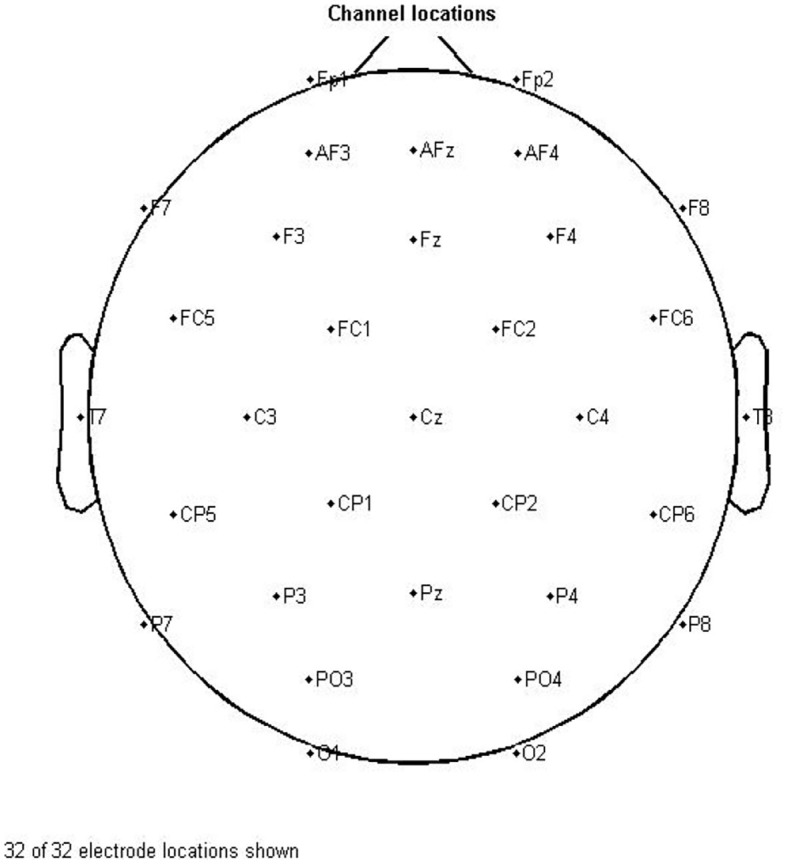
Channel locations for 32 electrodes.

The resting-state EEG data were recorded from healthy and RRMS subjects in a specialized clinical environment for 6 min using a 32-channel cap with a frequency rate of 512 Hz during eyes-closed with g.HIamp device from g.tec, which has the FDA and CE approval. Electrodes were positioned according to the international 10–20 system. The reference is the average signal recorded from the two electrodes placed on the mastoid bone on both sides of the skull. Table 3 and Figure 1 show the EEG recording setting and the location of channels. Before starting the data acquisition, the operator checked all channels for the best possible impedance and controlled the recording process. Although the data collection time was short, we checked the signals online and ensured that subjects did not show behavioral drowsiness during the data acquisition. During the data collection process, all subjects were in stable condition.

**Table 3:** EEG recording settings.

**Sampling rate**	**Input impedance**	**Electrode-Skin impedance**	**Number of channels**
512 Hz	>100 MΩ	<30 KΩ	32

### Preprocessing

**Figure 2:**
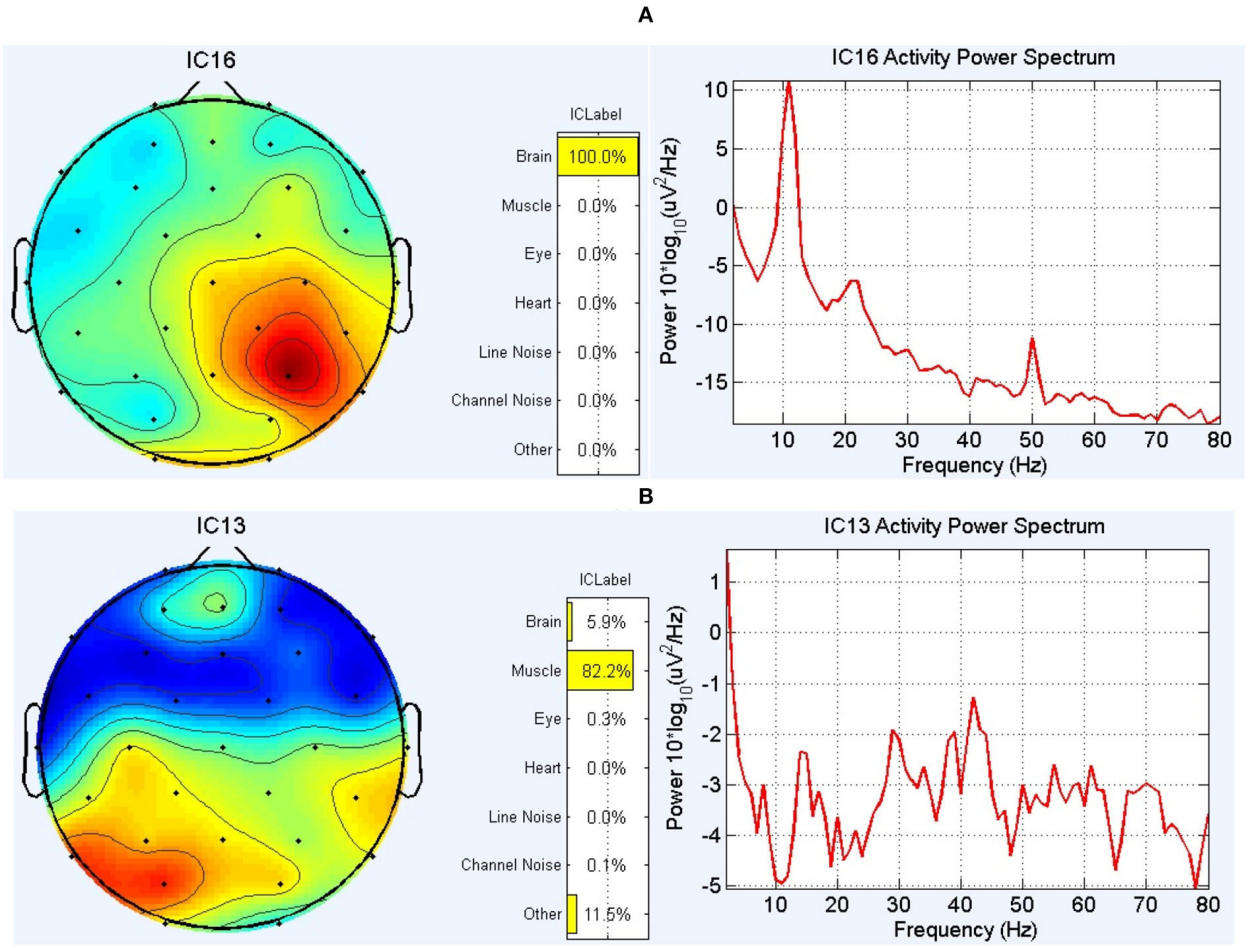
The results of the ICLABLE toolbox for two components in a subject. **(A)** An example of the topoplot and power spectrum of a component related to the activity of the brain and therefore was included in the calculation process. **(B)** An example of the topoplot and power spectrum of a component that is mainly related to muscle activity (according to 1-power-spectrum activity, 2-topoplot, and 3-location of this IC in the head model) and therefore was excluded from the data.

The EEG preprocessing was done using the EEGLAB toolbox (Delorme and Makeig, 2004). Frequencies lower than 0.5 Hz were removed using a high-pass FIR filter with an order of 826. Furthermore, the line noise was removed using the CleanLine toolbox (Mullen, 2012). In the next step, independent component analysis (ICA) (Comon, 1994) was performed to investigate components individually and remove those related to artifacts. Here, the original number of extracted independent components (ICs) equals the number of channels. To specify artifact-infected independent components, both the Multiple Artifact Rejection Algorithm (MARA) by the EEGLAB plugin (Winkler et al., 2011) and ICLABLE by EEGLAB plugin (Pion-Tonachini et al., 2019) were applied. The core of MARA is a supervised machine learning algorithm that learns from expert ratings of 1,290 components by extracting six features from the spatial, spectral, and temporal domains. In addition, features were optimized to solve the binary classification problem. By considering MARA and ICLABLE results simultaneously, the power spectrum of the extracted components was visually inspected, and then the non-brain ICs were discarded from the data (Figure 2). The DIPFIT toolbox (version 3.3) (Oostenvelt et al., 2003) and boundary element method (BEM) in the head model were used for source localization and demonstration of dipoles. DIPFIT can perform source localization by fitting an equivalent current dipole model using a non-linear optimization technique (Scherg, 1990) using a 4-shell spherical model (Kavanagk et al., 1978) or by using a standardized boundary element head model (Oostendorp and Van Oosterom, 1989). In this study, we wanted to investigate if RRMS disease affects the number and location of brain-related extracted ICs from EEG signals. After removing the ICs that were not brain-related, EEG signals from 32 channels were used after back projection for connectivity measurements.

### Connectivity measurements for network construction

In this study, the connectivity measures monitored the functional connectivity of the EEG signals. Here, the flow of information between EEG signals alongside their activity synchronization is investigated using weighted connectivity matrices. First, normalized DTF was calculated. The DTF can be defined as the total flow of information from component *j* to component *i* normalized by the total amount of inflow to component *i* and can represent the signal transfer in the network. In other words, DTF describes the causal influence of channel *j* on channel *i* at frequency *f*. The DTF method is based on the adaptive multivariate auto-regressive (AMVAR) model fitted to the EEG signal. DTF was designed to determine the interrelations between two signals in relation to all other signals in the analyzed system. One advantage of the DTF measure is that it is less likely to show spurious connections (Kamiński et al., 2001). It is defined using the elements *H*_*ij*_ of the transfer matrix of the MVAR model (Kaminski and Blinowska, 1991). The *H*(*f*) matrix is called a transfer matrix of the system, where ***f*** denotes frequency.


(1) $$\begin{array}{l} \gamma_{ij}\left(f\right)=\frac{H_{ij}\left(f\right)}{\sqrt{\sum_{k=1}^{M}|H_{ij}\left(f\right){|}^{2}}} \end{array}$$

The above equation shows the ratio between the inflow from component *j* to *i* to all the inflows to component *i*. DTF shows direct and cascade flows and has been proven as one of the valuable connectivity estimators in EEG-based studies (Astolfi et al., 2007).

The coherence is the frequency domain equivalent to the time domain cross-correlation function. Coherence between two signals measures the linear relationship between the two at a specific frequency. Coherence, *C*_*ij*_(*f*), quantifies the degree of functional association between two regions in the brain and is defined as below:


(2) $$\begin{array}{l} C_{ij}\left(f\right)=\frac{S_{ij}\left(f\right)}{\sqrt{S_{ii}\left(f\right)S_{jj}\left(f\right)}} \end{array}$$

Here, the *X*_*i*_(*f*) and *x*_*j*_(*f*) is the Fourier transforms of the time series *x*_*i*_(*t*) and *x*_*j*_(*t*) of channels *i* and *j*, then the cross-spectrum is defined as follows:


(3) $$\begin{array}{l} Sij\left(f\right)=<X_{i}\left(f\right)X_{j}^{*}\left(f\right)> \end{array}$$

The imaginary part of the coherence (iCoh) was measured to investigate the synchronization between the EEG channels. The imaginary part of coherence provides an excellent candidate for studying brain interactions (Sander et al., 2010; Pascual-Marqui et al., 2014; Sun et al., 2019). Although the usual magnitude and phase of coherency contain the same information as the real and imaginary parts, the cartesian representation is sometimes superior for studying brain interactions (Nolte et al., 2004). The iCoh can determine lag–lead relationships while invariant to linear instantaneous volume conduction.


(4) $$\begin{array}{l} i{Coh\left(f\right)}_{ij}=Im\left(C_{ij}\left(f\right)\right) \end{array}$$

The mentioned connectivity measures were calculated between the EEG channels and averaged for the frequency range of 0.5–45 Hz during the recorded time. In addition, the connectivity measures were calculated for individual frequency bands. The output of SIFT for each connectivity matrix is a 32^*^32/(frequency–range)/time matrix.

### EEG data analysis

A specific network was constructed for each subject in which the vertices of the networks were the EEG channels, and the edges were different connectivity measures, which we calculated between each pair of signals. The source information flow toolbox (SIFT) by EEGLAB (Mullen, 2010) was used for calculating the connectivity measurements.

#### Model order selection and validation

Akaike information criterion (AIC) was used for selecting the AMVAR model order (Akaike, 1974). Selecting the best possible model order helps to fit the data to the MVAR model while ensuring that the model presents valuable information, the noise is not mixed in the model and is stable. AIC chooses a model order that minimizes information criteria evaluated over a range of model orders. In the equation for this information criterion (equation number 5), the first term characterized the model's entropy rate, and the second term described the number of freely estimated parameters in the model. Here, $ln|\tilde{\sum \left(p\right)}|$ is the logarithm of the determinant of the estimated noise covariance matrix for the VAR model with the order of *P*. *M* is the number of components, and $\hat{T}=$ *TN* is the total number of samples. To check whether the VAR model was correctly fitted to the EEG data, three tests were implemented. First, we checked the stability and stationarity of the model. Second, we checked the model's residuals for serial and cross-correlation and eventually checked the model's consistency for each case. Finally, after fitting the estimated model order to the data, the model was validated by SIFT to ensure that it is useful for the available data.


(5) $$\begin{array}{l} AIC\left(p\right)=ln\left|\tilde{\sum \left(p\right)}\right|+\frac{2}{\hat{T}}PM^{2} \end{array}$$

#### Graph metrics

After calculating the mentioned connectivity measures, the minimum connected component (MCC) method was used to binarize connectivity matrixes (Vijayalakshmi et al., 2015). Implementing MCC can help select the more significant connections according to the nature of the network. Using MCC is more logical than manually choosing a threshold for each network without knowing what value is necessarily suitable. The MCC method has been used to investigate the cognitive load in the functional brain network and, therefore, can be helpful in this study according to the subjects (Vijayalakshmi et al., 2015). After network construction, graph theory metrics were compared to assess the characteristics of networks. The networks' diameter, global efficiency, and transitivity were calculated to investigate brain global network features. The network diameter can demonstrate the linkage of the network. At the same time, global efficiency and transitivity are useful features for showing the ease of information transactions between nodes. Table 4 contains a description of the investigated graph metrics in this study. The brain connectivity toolbox was used to calculate the network metrics (Rubinov and Sporns, 2010).

**Table 4:** Description of graph metrics.

**Metric**	**Description**	**Equation**
Transitivity	Transitivity is the ratio of triangles to triplets in the network	$T=\frac{3\times number\;of\;triangles\;in\;the\;network}{number\;of\;connected\;triples\;of\;nodes\;in\;the\;network}$
Global efficiency	Global efficiency is the average shortest path length in the network	$E_{global}=\frac{1}{N\left(N-1\right)}\underset{i\neq j}{\sum }\frac{1}{d_{ij}}$ *d*_*ij*_:*the shortest path length between node i and j*
Diameter	Diameter of a graph is the maximum eccentricity of any vertex in the graph. It is the greatest distance between any pair of vertices	$D=\max_{i,j}\left\{d_{i,j}\right\}$ *d*_*ij*_:*the shortest path length between node i and j*

## Results

After calculating the connectivity and construction of the brain network for each subject, we used the Shapiro-Wilk test to evaluate the normality of the distribution of results and the Wilcoxon rank-sum test to distinguish different features between the healthy and RRMS groups.

### Extracted dipoles and average power spectrum

**Figure 3:**
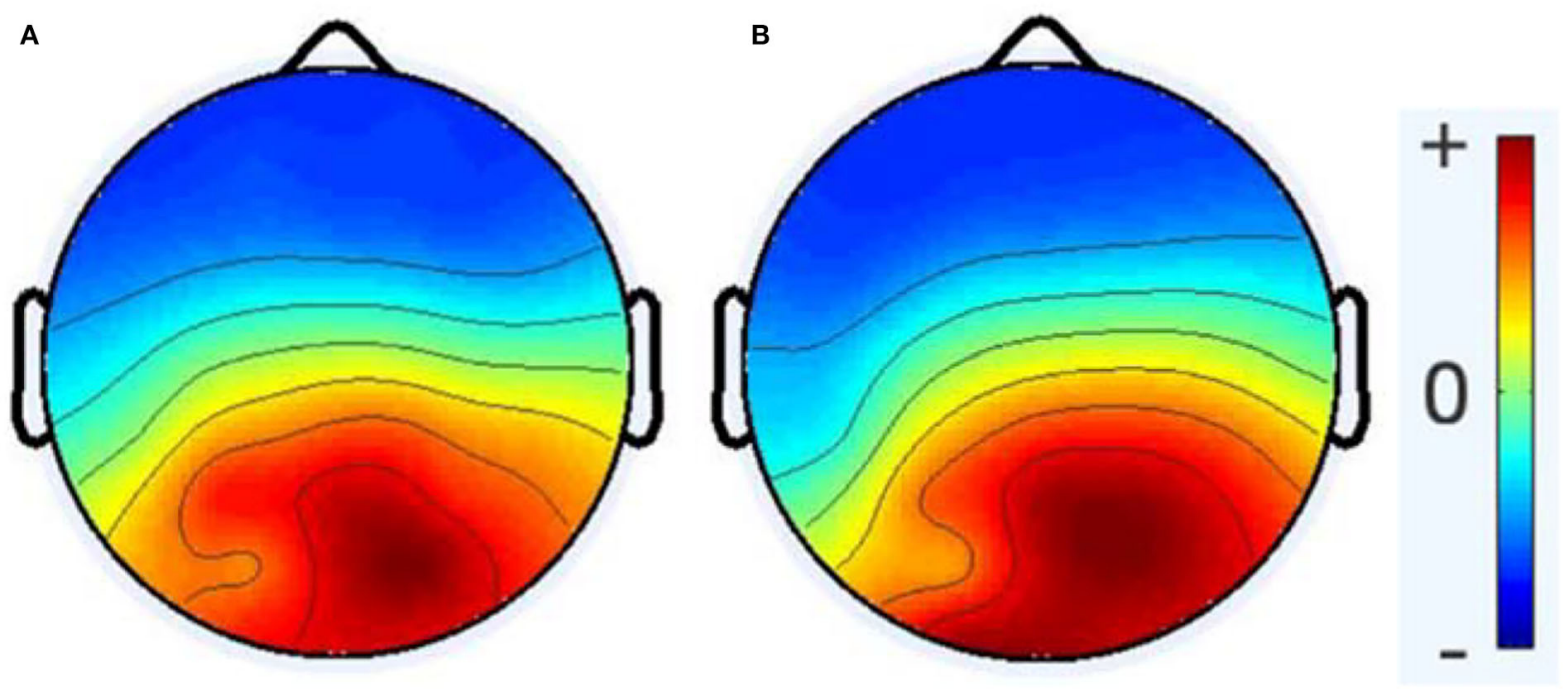
The topoplot of the average activity of ICA components for the entire frequency range (0.5–45 Hz) after preprocessing and excluding non-brain ICs, healthy group **(A)** and RRMS group **(B)**. Here, no significant differences were investigated between the two groups.

**Figure 4:**
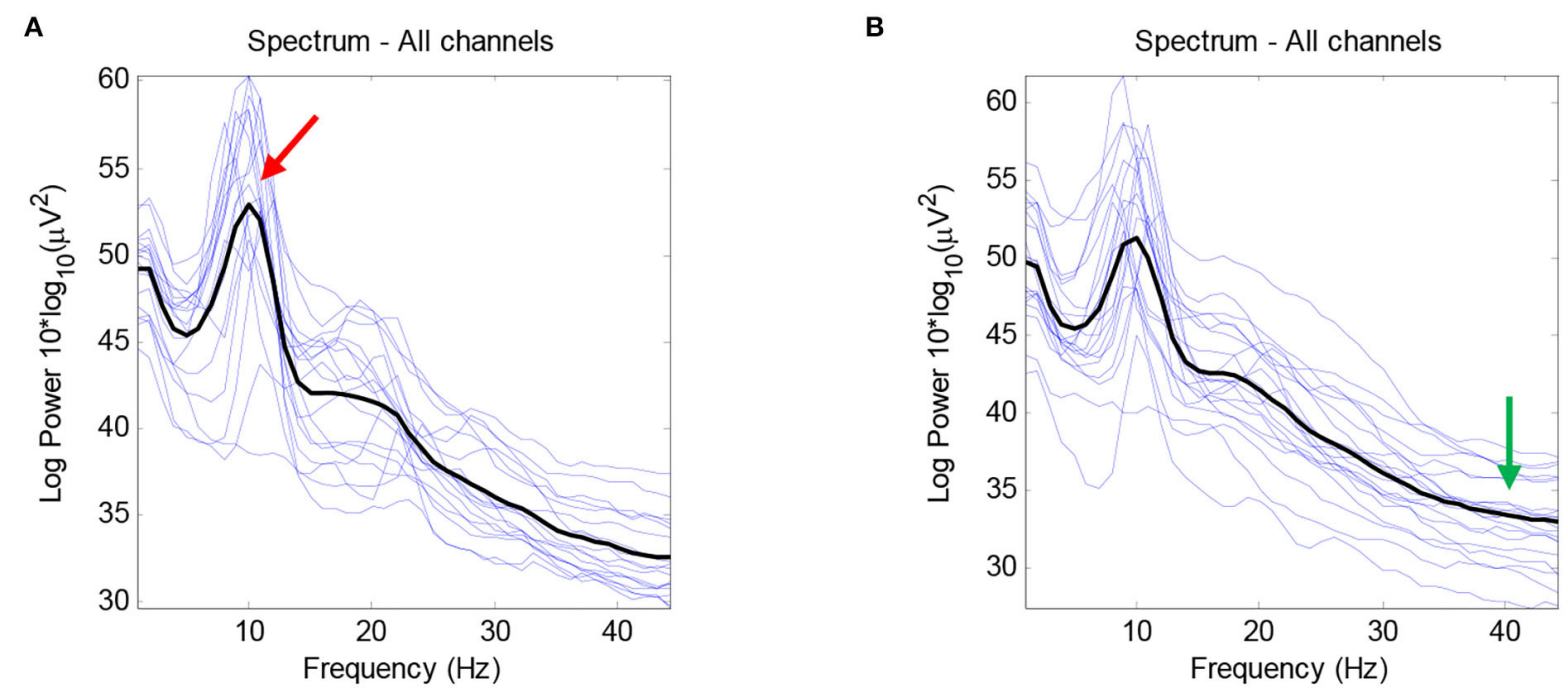
The average power spectrum plot after removing the artifacts for the healthy group **(A)** and the RRMS group **(B)** in black color using EEGLAB. The power spectrum plots for individual subjects are demonstrated in blue color. The peak in the alpha frequency band is higher in healthy subjects (red arrow), and the average gamma power is higher in patients with RRMS (green arrow). In this figure, the x-axis indicates the frequency, and the y-axis indicates the average power ($Log\;Power\;10*\log_{10}\left(\mu v^{2}\right)$).

The average number of ICs for the healthy group was 16.66, with a standard deviation of 3.97, while the average number of ICs for the RRMS group was 15.6, with a standard deviation of 3.574. According to the statistical analysis, no significant difference was observed between the number of brain-related ICs for the two groups. As Figure 3 demonstrates, no meaningful difference between the average activity of ICs for the entire frequency range was revealed between the two groups. The notable difference between the two groups is that the alpha peak's average power is higher in healthy cases than in patients with MS, with a *p*-value of 0.0411. In comparison, the average power of the gamma band is lower in healthy subjects, with a *p*-value of 0.0503. There were no other noteworthy differences in other spectral features for the alpha and gamma frequency bands. Furthermore, no meaningful differences were observed in other frequency bands between the RRMS and healthy groups. Figure 4 shows the average and individual subjects' power spectrum activity in sensor space for the two groups after excluding the components related to the artifacts.

### Connectivity graph metrics

To compare the linkage of the networks, the diameter of the networks is investigated. There is a significant difference between the two groups' network diameter for the DTF measure for the average of the frequency range. According to the Wilcoxon rank-sum test results, the network diameter for DTF is higher in RRMS cases in the alpha frequency band. The *p-*value for the DTF measure was 0.037. The diameter is also increased for the DTF connectivity measure for the average of the entire frequency range. The *p*-values for individual frequency bands are presented in Table 5.

**Table 5:** The *p*-value results of the Wilcoxon rank-sum test after comparing the graph features of calculated brain networks.

**Connectivity measure**	***P*-Value for diameter**	***P*-Value for global-efficiency**	***P*-Value for transitivity**
**(A) Total**		
DTF	**0.037**	0.621	0.835
iCoh	0.462	**0.044**	**0.027**
**(B) Delta band**		
DTF	0.722	0.658	0.881
iCoh	0.645	**0.0422**	**0.0316**
**(C) Theta band**		
DTF	0.447	0.534	0.959
iCoh	0.962	**0.0522**	**0.0337**
**(D) Alpha band**		
DTF	**0.0336**	0.794	0.917
iCoh	0.724	**0.0315**	**0.0535**
**(E) Beta band**		
DTF	0.149	0.361	0.373
iCoh	0.552	**0.0492**	**0.0518**
**(F) Gamma band**		
DTF	0.644	0.972	0.984
iCoh	0.551	0.448	0.691

**(A)** The results for the entire frequency band. **(B)** The results for the delta frequency band. **(C)** The results for the theta frequency band. **(D)** The results for the alpha frequency band. **(E)** The results for the beta frequency band. **(F)** The results for the gamma frequency band (The significant p-values are shown in bold).

**Figure 5:**
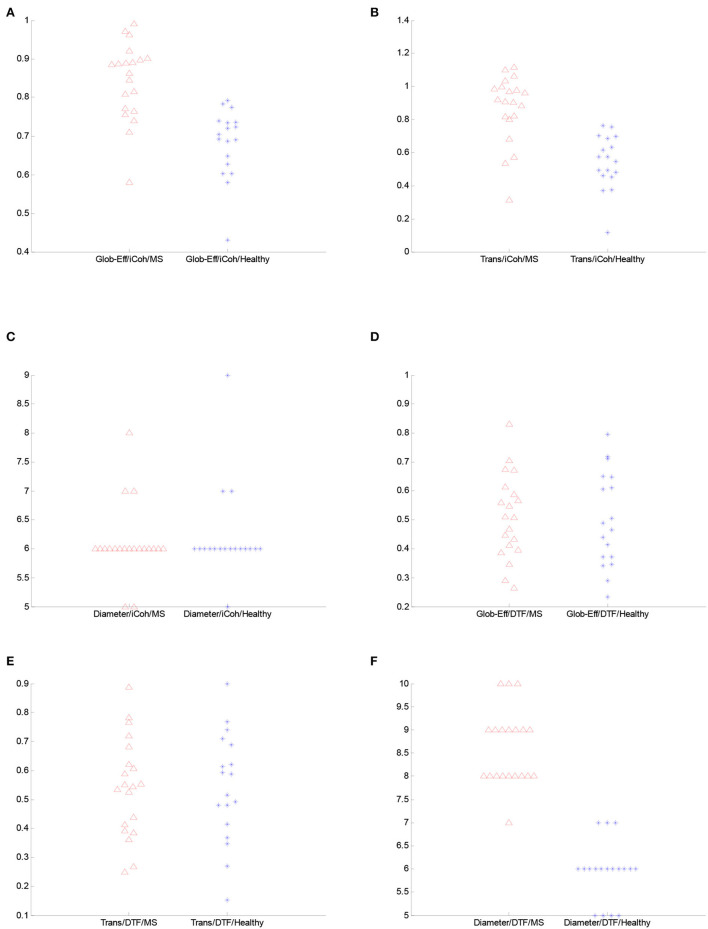
The figure illustrates the comparison of the spread plot of the graph features for the complete frequency range between the groups. **(A)** Global efficiency from iCoh. **(B)** Transitivity from iCoh. **(C)** Diameter from iCoh. **(D)** Global efficiency from DTF. **(E)** Transitivity from DTF. **(F)** Diameter from DTF.

Furthermore, both transitivity and global efficiency were calculated to investigate the brain network's ability to exchange data and the efficiency of signal transformation. There are significant differences between the two groups for the iCoh measure. According to the Wilcoxon rank-sum test results, both transitivity and global efficiency for iCoh are generally higher in patients with RRMS compared to healthy subjects. The *p-*value for the average of the entire frequency range global efficiency was 0.044, and for transitivity was 0.027. Moreover, transitivity and global efficiency were higher for the iCoh measure in the delta, theta, and beta frequency bands. Figure 5 demonstrates the values of graph metrics calculated for the connectivity measures used in this study. Here, we can see a clear separation in the distribution of global efficiency and transitivity based on the iCoh measure and the diameter based on the DTF measure for the frequency range of 0.5–45 Hz between the groups.

## Discussion

This study demonstrated a lower amplitude in the alpha peak and a higher amplitude in the gamma band for patients with RRMS than healthy subjects. In the previous studies, irregularities in individual frequency bands' activity, such as alpha, beta, and gamma, have been demonstrated. These studies have shown a lower amplitude for the alpha frequency band (Babiloni et al., 2016). The resting-state eyes-closed EEG rhythms exhibit a condition of relative relaxation and inhibition of brain mechanisms subserving the interaction with the external world. A complex pattern of parallel neural networks generates these rhythms in which the integrative activity of the neurons is characterized by a synchronous oscillatory mode across neural assemblies (Nunez, 1989; Başar, 2012). A pathological decrease in the resting-state alpha frequency band in patients with MS probably indicates a pathological desynchronization of diffuse neural networks regulating the fluctuation of cortical arousal and tonic alertness. When a person is involved in a situation that captures his awareness or implies various actions, alpha and low-frequency beta rhythms reduce amplitude, which shows a desynchronization. Here, these slower oscillations are replaced by fast oscillations at high-frequency beta and gamma (Pfurtscheller and Da Silva, 1999).

Network diameter is the shortest distance between the two most remote nodes in the network. A higher diameter implies a less-linked network. According to the DTF connectivity calculations, which designate the level of signal transformation and association between different channels, the linkage in RRMS subjects is lower than in the healthy group. These results are possibly the effect of axonal demyelination and the existence of lesions in the neural network, specifically in cortical and subcortical regions, which not only can manipulate the rate of signal transfer but also, in more severe conditions, can lead to the complete loss of communication between different regions. According to previous studies, a predominant pattern of decreased global connectivity due to acute neuroinflammation (Kocevar et al., 2016) or increased lesion load can be associated with the disease course (He et al., 2009). Several fMRI studies have shown reduced coherence in resting-state networks in MS cases (Rocca et al., 2012; Sbardella et al., 2015). Furthermore, previous studies have shown a correlation between the cortical and subcortical damage that causes cognitive impairments in MS disease with the decrease in the coherence connectivity in the alpha frequency band. In previous EEG-based studies, additional information from MRI has shown a correlation between the lesion load and the abnormal characteristics of functional brain networks related to the cognitive impairments in MS (Leocani et al., 1998, 2000).

In addition to the network linkage based on DTF, the results of this study showed significant differences in the level of global network synchronization between the two groups. According to the iCoh connectivity measure, the level of transitivity and global efficiency is generally higher in RRMS cases compared to normal subjects for the iCoh measure for the average frequency range and delta, theta, and beta frequency bands. An increase in transitivity represents a cost-efficient organization principle of the brain with increased local cliquishness (Newman and Park, 2003). Furthermore, the increase in global efficiency indicates that information transfer across the whole brain is more efficient (Latora and Marchiori, 2001). Therefore, the higher transitivity and global efficiency for iCoh indicate a higher global synchronization of the brain network in MS cases. Previous research on functional connectivity changes using MEG resting-state data indicated an increase in synchronization in the theta, lower alpha, and beta bands (Schoonheim et al., 2013).

The increased synchronization in the mentioned frequency bands and differences between DTF and iCoh measures needs further study to show what exactly it means. However, the global synchronization increase, especially in the alpha frequency band, can represent the compensation reaction of the brain in MS cases. Although in the previous studies, there have been discrepancies between the results of fMRI and MEG studies that can be explained by the fundamental differences between fMRI and MEG since fMRI cannot detect high-frequency oscillations. Previous fMRI studies have shown that functionally linked brain regions compensate for the structural damage caused by MS (Droby et al., 2016). fMRI studies have shown that when MS causes damage in a specific area, new areas are sometimes recruited to perform the tasks previously performed by the damaged area. These studies have elucidated the role of cortical reorganization in suggestive MS cases (Rocca et al., 2003; Rocca and Filippi, 2007). Furthermore, another study has shown the compensatory cortical activation during the earliest stage of MS (Audoin et al., 2003). Compensation reaction and compensation failure have been studied in MS disease for cognitive tasks during task-directed studies (cognitive compensation failure in MS). The compensation reactions of the brain have been observed in other studies, and it is not limited to MS cases (Klöppel et al., 2009; Dixon et al., 2013). A recent fMRI study showed continuous functional reorganization between specific brain regions such as fronto-cerebellar connections, which were closely related to clinical performance, indicated by increasing effective connectivity over time in MS cases, which was not noticeable in healthy cases (Fleischer et al., 2020). These results can show the role of functional reorganization in compensating for the damage and abnormalities in these cases. Moreover, results from a recent study showed a correlation between cortical plasticity and cognitive impairment in which the plasticity is reduced in cases with cognitive impairment compared to the cases with preserved cognitive function, and second, generally, the RRMS group did not show reduced cortical plasticity compared to the control group (Balloff et al., 2022). Although this study used a specific approach to investigate cortical reorganization and does not illustrate the difference between RRMS patients with normal cases, it shows that in RRMS cases without cognitive impairment, brain cortical reorganization compensates for structural damage. Considering the condition of RRMS cases in this research, the results of our study suggest that we can detect brain cortical reorganization even during the resting state while there is no specific cognitive task using surface EEG. One possible way to further investigate resting-state EEG capability for monitoring cortical reorganization and its relation to cognitive functioning is to perform a comprehensive cognitive test while recording EEG in addition to the resting-state EEG. In addition to the increase in global efficiency and transitivity for the iCoh in the alpha frequency band, the global increase in synchronization for the average of the entire frequency range might be related to damaged inhibitory neurons in MS disease (Schoonheim et al., 2013), which can lead to a higher possibility of firing rate in the network. However, it is important to remember that excitatory neurons can be damaged as well as inhibitory neurons, and further investigations are needed to explain the global increase in synchronization in RRMS subjects.

## Conclusion

The application of EEG-based connectivity techniques in patients with MS has undoubtedly helped to improve our understanding of the mechanisms responsible for clinical disability and cognitive impairment in this condition. In this study, we used EEG resting-state signals and calculated the connectivity between EEG channels to investigate the functional brain network's global characteristics in patients with RRMS. This study's results demonstrated significant changes in brain functional network characteristics in patients with RRMS that are possibly related to damaged cortico-cortical and cortico-subcortical connections that lead to cognitive impairments in patients with MS. To have a better understanding of the relationship between the number of lesions and their location in the brain to the compensation reaction of the brain's functional network related to cognitive impairments, further studies are necessitated.

## Limitations of the study

Regarding the limitations of this study, there is room for improvement in the data we recorded. First, the number of channels can be higher, which helps to have better spatial resolution and source separation. However, it needs to be mentioned that a higher number of channels increases the preparation time, making the patients uncomfortable. The second point which can be improved is that we recorded data from only the relapsing-remitting subgroup and each patient in a single session. By increasing the diversity of the patients and including other subgroups as well, and recording data from the same subjects several times, not only we can compare various stages of the disease better, but also, we can assess the effectiveness of the treatment methods with higher confidence in the future. However, it must be mentioned that other subgroups have a delicate situation due to the major development and progression of their disease, so gathering data from these groups is much more complicated and needs full permission from clinical doctors and the ethics committee. Finally, it is expected that, like any other research work, in terms of the method and pipeline we used, there is always room for improvement, which is something we look forward to in future projects related to this topic.

## Data availability statement

The raw data supporting the conclusions of this article will be made available by the authors, without undue reservation.

## Ethics statement

The studies involving human participants were reviewed and approved by Iran Medical University. The patients/participants provided their written informed consent to participate in this study.

## Author contributions

SS: analysis and interpretation of data and drafting the manuscript. MM: conception and design of study and revising the manuscript critically for important intellectual content. Both authors contributed to the article and approved the submitted version.

## Conflict of interest

The authors declare that the research was conducted in the absence of any commercial or financial relationships that could be construed as a potential conflict of interest.

## Publisher's note

All claims expressed in this article are solely those of the authors and do not necessarily represent those of their affiliated organizations, or those of the publisher, the editors and the reviewers. Any product that may be evaluated in this article, or claim that may be made by its manufacturer, is not guaranteed or endorsed by the publisher.
